# The Relationship between Supportive Care Needs and Health-Related Quality of Life in Cancer Patients

**DOI:** 10.3390/healthcare11152161

**Published:** 2023-07-29

**Authors:** Andreas Hinz, Antje Lehmann-Laue, Diana Richter, Michael Hinz, Thomas Schulte, Evelyn Görz, Anja Mehnert-Theuerkauf

**Affiliations:** 1 Department of Medical Psychology and Medical Sociology, University of Leipzig, 04103 Leipzig, Germanyanja.mehnert@medizin.uni-leipzig.de (A.M.-T.); 2 Rehabilitation Clinic Bad Oexen, 32549 Bad Oeynhausen, Germanygoerz.evelyn@gmail.com (E.G.)

**Keywords:** supportive care needs, quality of life, importance, satisfaction, cancer

## Abstract

The aim of this study was to analyze the relationship between quality of life (QoL) and supportive care needs (SCNs) in cancer patients. It is difficult to relate SCNs to detriments in QoL since SCNs and QoL assessment tools generally comprise different dimensions that cannot be directly related to each other. Therefore, we developed a short questionnaire with eight dimensions for uniformly measuring SCNs, QoL, and the subjective importance of these dimensions. A total of 1108 cancer patients with mixed diagnoses assessed eight dimensions of health-related QoL concerning SCNs, satisfaction, and importance. Among the eight dimensions of QoL, *physical functioning* received the highest SCN assessments (M = 3.4), while *autonomy* (M = 20.7) and *social relationships* (M = 1.88) were the dimensions with the lowest SCN mean scores on the 1–5 scale. For each of the eight dimensions, high levels of SCNs were reported by those patients who had low levels of satisfaction with that dimension (*r* between −0.32 and −0.66). The subjective importance of the dimensions was not consistently correlated with SCNs (*r* between −0.19 and 0.20). Females reported higher SCNs than males in six of the eight specific dimensions. Patients with prostate and male genital cancers reported the lowest SCNs. These results suggest gender-specific SCN patterns that warrant further exploration. This study highlights the value of a unified assessment instrument for SCNs and QoL, providing a robust basis for future cancer care strategies.

## 1. Introduction

In recent decades, quality of life (QoL) has gained increasing importance in oncological research and practice, and multiple studies have confirmed detriments in QoL in cancer patients [1,2,3]. There is also a large body of research on supportive care needs (SCNs) experienced by cancer patients [4]. Several studies have analyzed associations between the dimensions of QoL and SCNs. High levels of SCNs are generally associated with low levels of mental health and low levels of QoL [5,6,7,8,9,10,11]. More specifically, the SCN dimension “Physical and daily living needs” is more strongly correlated with the physical component of QoL, whereas the SCN dimension “Psychological needs” shows stronger correlations with the mental health component [5]. A further study with cancer patients found correlations between the five dimensions of the Supportive Care Needs Scale SCNS-SF34, and distress, anxiety, and depression with coefficients between 0.18 and 0.60, the highest of those being that between the psychological component of the SCNS-SF34 and anxiety (*r* = 0.60) [6]. A further study conducted with breast cancer patients also found the highest association between anxiety and the psychological SCNS-SF34 dimension with *r* = 0.37 [12]. 

Conducting research on the relationship between SCNs and QoL is, however, made very difficult by the fact that QoL questionnaires and instruments for measuring SCNs have been developed largely independently and thus feature differently designed dimensions from one questionnaire to the next. This complicates the analysis of their relationships. Frequently used instruments for measuring QoL in cancer patients are the EORTC QLQ-C30 [13], which has 5 functioning scales and 10 other scales, and the SF-36 [14] comprised of eight dimensions, including two factors of higher order. Among the instruments designed to measure SCNs [4], those frequently used are: the Supportive Care Needs Survey instrument SCNS-SF34 [15] comprising five scales (health system and information needs, psychological needs, physical and daily living needs, patient care and support needs, and sexuality), the Cancer Survivorship Unmet Needs tool CaSUN [16,17] comprising five dimensions (information, comprehensive cancer care, existential survivorship, QoL, and relationships), and the Cancer Needs Questionnaire [18,19] comprising five scales (psychological, health information, physical and daily living, patient care and support, and interpersonal communication needs). However, it is not possible to precisely compare SCNs and QoL for specific dimensions of QoL because of the diversity of the dimensions used in both types of questionnaires. One recent study defined three domains of distress and reported correlations between the distress perceived in those domains and the corresponding requests for support, with coefficients between 0.67 and 0.69 [11]. However, these domains were relatively broad. For this reason, we conducted a study with a new questionnaire comprising eight specific dimensions in which the QoL and SCN dimensions are identical. 

In addition to satisfaction with dimensions of QoL and corresponding SCNs, the perceived subjective importance of these dimensions of QoL and SCNs is relevant. We suppose that SCNs are high when there are detriments in a dimension and when this dimension is important to the patient. QoL questionnaires generally do not take into account the subjective importance of the dimensions of QoL; it is implicitly assumed that all the dimensions have the same meaning for all patients. There are some questionnaires that also consider the subjective importance of dimensions of QoL, e.g., the Schedule for the Evaluation of Individual Quality of Life (SEIQoL) [20] and the Patient Generated Index (PGI) [21]. Some examinations investigated the association between satisfaction with QoL dimensions and the subjective importance of those dimensions [22,23,24], but, in the context of SCNs, the problem remains that those instruments’ dimensions are also difficult to compare to the dimensions of SCNs. 

Cancer patients perceive changes in functioning abilities and in symptom burden due to the disease and its treatment [25,26,27]. These changes can lead to changes in their frames of reference when evaluating QoL dimensions, a phenomenon referred to as response shift [28,29]. While changes in QoL have been extensively studied in oncological research, changes in the subjective importance are under-researched. 

A further issue is sex and age differences in the SCNs. Younger patients reported more SCNs than older ones [6] and females had a higher desire for support than males [6,18] with the exception of the domain sexuality, where the males wished for more support [6]. However, other studies did not detect statistically significant sex differences in the SCNs [30,31].

Given the gaps in the current literature, our study aims were (a) to identify the dimensions with high SCNs in a large sample of cancer patients (*n* > 1000), (b) to analyze the relationship between SCNs and satisfaction and importance assessments, including the perceived changes in satisfaction and importance of these dimensions, and (c) to test the impact of sex, age, and tumor type on SCNs.

## 2. Materials and Methods

### 2.1. Sample of Cancer Patients

Between September 2020 and May 2021, the study participants were recruited in a German oncological rehabilitation clinic. In Germany, most cancer patients are offered the opportunity to take part in a rehabilitation program to help restore their physical and psychosocial functioning after cancer treatment. Inclusion criteria for this study were: a proven cancer diagnosis, sufficient command of the German language, absence of severe cognitive impairment, and age 18 years and above. The study was approved by the Ethics Committee of the University of Leipzig (approval number 013/19-ek). Written informed consent was obtained from the participants after they were given a full explanation of the purpose and nature of the data collection and storage. Finally, a total of 1547 patients were asked to participate and 1108 (71.6%) of them agreed to take part in the study. These patients completed the questionnaires during their stay in the rehabilitation clinic. 

### 2.2. Instruments

#### Questions on HRQoL

Because no instrument was available for the simultaneous and joint assessment of QoL, SCNs, and subjective importance, we defined eight dimensions of health-related QoL, based on the dimensions of other frequently used instruments such as the EORTC QLQ-C30 [13] and the SF-36 [14]: *physical functioning, autonomy, emotional stability, cognitive functioning, social relationships, vitality, absence of pain,* and *sleep quality*. In addition to these eight specific dimensions, the participants were asked to assess their global health state concerning importance and satisfaction. The first five of the eight dimensions (from *physical functioning* to *social relationships*) were taken from the functioning scales of the EORTC QLQ-C30. *Vitality* was taken from the SF-36. This scale can be considered the opposite of *fatigue* which is also a scale of the EORTC QLQ-C30. Pain is also a dimension of both the EORTC QLQ-C30 and the SF-36 indicating one component of (low levels of) QoL. Here we use the term “*absence of pain*” instead of “*pain*” (in contrast to the SF-36) so that high scores of this scale correlate positively with high degrees of HRQoL for all of the eight scales. *Sleep quality* is a scale of the EORTC QLQ-C30 but not of the SF-36. This dimension was also included because of its special importance for cancer patients [32,33].

Each of these nine dimensions (one general dimension and eight specific dimensions) had to be evaluated concerning three perspectives: “To what degree do you wish support in (e.g., physical functioning)?”, “How satisfied are you with your (e.g., physical functioning)?”, and “How important is (e.g., physical functioning) for you?” 

For each of the questions there were five answer categories: “To what degree do you wish support…” (no support at all, …, a lot of support), “How satisfied are you with your…”: (very dissatisfied, …, very satisfied), and “How important is…”: (not important, …, very important).

The participants were also asked to evaluate changes in their satisfaction with the dimensions and in the importance of those dimensions to them on a five-point scale with the following response options. The questions and possible responses were: “How did your satisfaction (e.g., with physical functioning) change from the time before being diagnosed and now” (I became much more dissatisfied, …, much more satisfied), and “How did the importance (e.g., of physical functioning) to you change from the time before being diagnosed and now” (it became much more unimportant, …, much more important).

### 2.3. Statistical Analysis

The associations between the SCNs, satisfaction ratings, and importance ratings were calculated with Pearson correlations. The effects of age group and sex on SCNs were statistically tested with two-way ANOVAs, and the effect of tumor type on SCNs was tested with one-was ANOVAs. Effect sizes (Cohen’s *d*) were calculated to indicate the magnitude of the sex differences in SCNs. All statistics were performed with SPSS, version 27.

## 3. Results

### 3.1. Sample Characteristics

The questionnaire was completed by 404 male and 704 female patients (*n* = 1108). Table 1 presents further characteristics of the sample of participants. The mean age was 53.1 ± 14.6 years (range: 18–88 years). 

### 3.2. Mean Scores for SCNs, Satisfaction, and Importance

**Figure 1:**
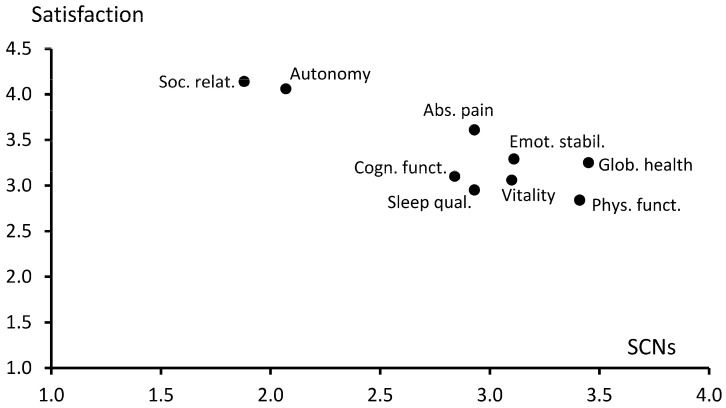
Relationship between SCNs and satisfaction mean scores.

Table 2 shows the mean scores for the eight dimensions of QoL from five perspectives: SCNs, satisfaction, importance, change in satisfaction, and change in importance, see also Figure 1. The highest levels of SCNs were found for *physical functioning* (M = 3.41) and *global health* (M = 3.45), while the lowest scores were found for *autonomy* and *social relationships*. In contrast to that, the highest satisfaction was found for *autonomy* and *social relationships*, and low satisfaction ratings were recorded for *physical functioning* and *sleep quality*. *Autonomy* was the dimension with the highest perceived importance (M = 4.51). 

Concerning changes in satisfaction, the patients perceived detriments in all dimensions except *social relationships* and the most pronounced negative changes were found in the dimension *physical functioning* (M = 2.02). All dimensions gained in importance after the occurrence of the disease, with the most pronounced increases being observed for *global health* (M = 4.39) and *emotional stability* (M = 4.04).

Considering these results together, there is a certain trend that dimensions with high satisfaction mean scores (in particular *autonomy* and *social relationships*) are characterized by low SCNs, while on the other hand the dimension with the lowest satisfaction (*physical functioning*) shows the highest demands for SCNs.

### 3.3. Correlations between SCNs, Satisfaction, and Importance of the Dimensions

Table 3 presents the correlations between SCNs and other aspects of the dimensions. For each dimension of QoL, SCNs were negatively associated with satisfaction, with coefficients ranging from −0.32 to −0.66. The strongest associations were found for *sleep quality* (*r* = −0.66) and *cognitive functioning* (*r* = −0.58).

The subjectively experienced importance of the dimensions was only weakly correlated with SCNs, with coefficients between *r* = −0.19 and *r* = 0.20.

The associations between SCNs and changes in satisfaction were in a similar sequence to those of the SCN–satisfaction correlations, with somewhat weaker correlations; the association was also strongest for the dimension of *sleep quality* (*r* = −0.48). In contrast to that, changes in importance were more strongly associated with SCNs than the importance ratings themselves, with coefficients between 0.21 and 0.48. Patients who perceived an increase in the importance of *sleep quality* reported more SCNs than those with no such increase in the importance of *sleep quality* (*r* = 0.48).

Taking these results together, the negative association between SCNs and satisfaction ratings that could already be seen in the sequences of the mean scores (Table 2) could be confirmed for each of the dimensions separately with relatively strong correlation coefficients, while the correlations between SCNs and importance scores and change scores were less clear.

### 3.4. Sex and Age Differences in SCNs

Table 4 shows that females reported higher levels of SCNs than males in six out of the eight specific dimensions of QoL, with the most relevant sex differences occurring in the *emotional stability* (*d* = 0.35) and *cognitive functioning* (*d* = 0.28) dimensions. *Autonomy* and *social relationships* were the only dimensions in which the females’ SCNs were lower than those of the males. Concerning age differences in the SCNs, the results were also mixed. With the exception of two dimensions, *autonomy* and *social relationships*, younger patients reported higher levels of SCNs than older patients did. As was the case in sex differences, the highest age differences were found for *emotional stability* (*d* = −0.48) and *cognitive functioning* (*d* = −0.34). Table 4 also shows that there were no significant ANOVA interaction effects between sex and age.

Table 4, lower part, presents the SCN mean scores, broken down by tumor localization. Regarding global health, two groups showed low levels of needs: prostate cancer patients and patients with male genital cancers (M = 3.11 and M = 3.14), while the mean scores of all other groups were relatively similar, between M = 3.47 and M = 3.58. The comparison between the mean scores of the prostate cancer patients and the mean score of the total of males in Table 4 shows that prostate cancer patients perceived fewer SCNs in all eight dimensions of QoL in comparison with the other male patients.

In summary, neither sex nor age showed that one sex or age group consistently indicated higher SCNs in all of the eight dimensions than the other group.

## 4. Discussion

The central objective of this study was to examine the relationship between SCNs and QoL for different dimensions of QoL. All correlations were negative, indicating that greater detriments in QoL dimensions are associated with higher levels of SCNs in those dimensions. This general association between detriments and SCNs has also been found in other studies [5,6,34]; however, it is not possible in those cases to analyze the relationships for the dimensions precisely since the dimensions of SCNs (e.g., assessed with the SCNS-SF34) did not precisely correspond with the dimensions of the QoL assessment instruments. In our study, we can compare the eight dimensions of QoL with regard to this relationship separately.

The negative association between SCNs and satisfaction was varied across different dimensions. *Sleep quality* showed the strongest correlation (*r* = −0.66), while the weakest correlations were obtained for *physical functioning* (*r* = −0.32) and *social relationships* (*r* = −0.34). Patients with sleep problems appear to trust in supportive care techniques for these problems (e.g., helpful medication), while good sleepers do not see a necessity for support, both factors that lead to the strong correlation. The interpretation is more difficult in the *physical functioning* and *social relationships* dimensions, where the associations were weakest. *Physical functioning* was the dimension with the highest SCN mean score (M = 3.41) and *social relationships* had the lowest SCNs mean score (M = 1.88). Obviously not only patients with physical problems but all patients seek help in improving their physical functioning, which results in the relative low correlation. *Social relationships* are not unimportant; in fact, they received the second highest importance rating. That said, it is possible the patients consider social relationships beyond the scope of SCNs and a matter of their own responsibility, which may explain the low level of SCNs in this dimension and the low association with the satisfaction scores. In a recent psycho-oncology study [11], the correlations between three domains of distress (emotional, practical, and physical–functional) and the corresponding requests for help were shown. In this study, the three correlations were very similar, ranging from 0.67 to 0.69. The reason for the similarity of the correlations in this study, in contrast to the results of our study, might be the relatively broad dimensions. For example, the domain of sleep quality is subsumed under physical–functional, whereas our eight dimensions were more specific.

It is interesting to note that, while *cognitive functioning* was only rated as having moderate subjective importance, the association between SCNs and satisfaction with *cognitive functioning* is relatively strong (*r* = −0.58) for that dimension, meaning that patients who perceive cognitive impairments experience a great need for help in that area. Such results can only be obtained when the assessment tools for SCNs, satisfaction, and importance can be uniquely matched, as is the case in our methodology.

The subjective importance of QoL dimensions proved to be largely independent from SCNs; the correlations ranged between −0.19 and 0.20. This means that physicians should not only ask whether a dimension is important to a person or not, but also whether the patient has a wish for support in that area. Physicians should be aware that a patient’s assessment of unmet SCNs may differ from the perception of their oncologists [35]. This should also be acknowledged when physicians and patients come to a common decision about the goals of the treatment [36]. Moreover, SCNs are not only relevant for patients; we also find supportive care needs in cancer care providers and in the patients’ families, in particular in the context of palliative medicine [37,38,39].

In our study, we assessed SCNs without a deeper specification of their nature; we did not ask whether the needs were unmet or not [16,40], and we also did not ask whether there were unexpressed needs among the SCNs. A study with palliative cancer patients [41] considered four categories of SCNs: no problems, problems but no need, met need, and unmet need. Most patients reported no problems with concentration (84%) in that study but among the patients with concentration problems the frequency of unmet needs (70%) was the highest. A recent examination with cancer patients found that 24% of the patients reported that they had unexpressed needs, and having unexpressed needs was associated with lower mental health and lower levels of QoL [40,42].

In addition to the satisfaction and importance ratings, we also included assessments of changes in satisfaction and importance since the beginning of the disease. With the exception of *social relationships*, the means of all satisfaction change scores were below 3 on the 1–5 scale, indicating a decrease in satisfaction, and all importance change scores were above 3, indicating an increase in their perceived importance since the time of diagnosis. The increase in satisfaction with *social relationships* may possibly be due to the fact that *social relationships* were intensified during the illness process, and that these social relationships were experienced as being reliable and helpful.

Detriments (negative changes) in participants’ satisfaction with QoL domains were associated with high levels of SCNs (see Table 3), with correlations between *r* = −0.15 (*social relationships*) and *r* = −0.48 (*sleep quality*), once more indicating the need for help in the domain of sleep. Concerning changes in the subjective importance of the dimensions, we found positive correlations between all the dimensions and the SCNs ranging from *r =* 0.21 (*physical functioning*) and *r =* 0.48 (*sleep quality*). While the direct assessment of the subjective importance of the dimensions was not clearly associated with SCNs (*r* between −0.19 and 0.20), the changes in their importance to the patients underline the association between importance and SCNs in the longitudinal context.

Females reported more SCNs than males did in six of the eight dimensions. The highest sex differences were found for the psychological areas of *emotional* and *cognitive functioning*, which is in line with the literature [6,18,43]. Concerning *autonomy* and *social relationships*, males reported more SCNs than females. One possible reason is that *autonomy* is perceived as being especially relevant for males and that this implies that they seek help in this area more than females do. *Social relationships* may generally have a greater relevance for females than for males, but females probably trust in their own abilities to manage these relationships and thus may not see themselves as needing help in this context, a possible explanation for the low level of women’s reported need for help in the *social relationships* dimension.

Concerning age differences, younger patients reported higher levels of SCNs than older patients in most dimensions. Two studies also investigated age differences in SCNs, using the SCNS-SF34. One of these studies, performed in Hong Kong [5], also found statistically significant age differences with higher levels for younger patients in all five dimensions (effect sizes between d = 0.28 and 0.45), while the other study, conducted in Malaysia [44], failed to detect such age differences in four of the five dimensions. Cultural differences in reporting SCNs may contribute to such different results. We see two possible reasons for the higher levels of SCNs among younger patients in our study. First, their disease burden is perceived as stronger in comparison with the older patients which is also reflected in the higher levels of anxiety and distress levels reported by younger patients in comparison with their healthy peers [45]. The second possible reason is that younger patients may be more willing to admit their psychosocial wishes and needs, that they know more options of psychosocial support, and that they think that it is appropriate to seek help in the context of the health care system. *Emotional stability* and *cognitive functioning* were the two dimensions with the highest age differences. These are also the dimensions with the most pronounced sex differences. Sex and age are confounded to a certain degree (e.g., older males with prostate cancer), but the ANOVA results show that the interactions between sex and age were small, and that both sex and age effects are statistically significant for *emotional stability* and *cognitive functioning*. The presence of these age and sex differences implies that supportive cancer care should consider the needs of female patients in particular to narrow the gender disparity in cancer burden, and that younger patients should be given special attention [46,47]. For males, the area of autonomy should be given particular consideration.

For most cancer localizations we found SCN mean scores of about 3.5 on the 1–5 scale and there was no cancer category with markedly heightened SCNs in comparison to the other categories. The tumor types with the lowest SCNs were prostate cancer (M = 3.11) and male genital cancer (M = 3.14), though one must take into consideration here that tumor type and sex are confounded. A more detailed analysis homing in on the specific contributions of cancer type and confounding factors such as sex or age would help clarify the influencing factors for SCNs; however, such analyses would be beyond the scope of this paper which is focused on the relationship between SCNs, satisfaction, and importance.

Some limitations of this study should be mentioned. The sample consisted of patients taking part in a rehabilitation program. Therefore, the sample is not representative of all cancer patients and future studies should examine whether the results can be replicated in other settings. Furthermore, it is unclear to what degree results obtained in a Western country may be generalized worldwide; unmet needs proved to be more frequent in Low and Medium Income Countries than in High Income Countries [48]. As already mentioned above, we did not differentiate between met and unmet needs.

QoL, satisfaction, and SCNs were assessed with only one item for each dimension. Instruments with more items per dimension such as the SCNS-43 will probably be more reliable in assessing the scores of the individuals. However, single-item measures were used in many studies dealing with the subjective importance of domains, and these single-item measures proved to be meaningful and reliable, even in the evaluation of complex constructs such as well-being [49]. Since in our study all of the dimensions were assessed with single items in a uniform way, the degree of reliability will be similar for the dimensions and the effects of the different dimensions can therefore by fairly compared with one another.

## 5. Conclusions

Health care providers should note that limitations in certain aspects of QoL do not equate to a need for support in these areas. In addition to the areas generally considered, such as pain and physical functioning, other areas proved to be essential. Sleep problems and cognitive problems should be considered because of the strong correlations between SCNs and satisfaction in these areas. The completion of questionnaires on health-related QoL is an important means of capturing patients’ subjective assessment of their personal situation but it should be supplemented by explicit questions about SCNs.

The instrument for assessing SCNs, QoL, and subjective importance proved to be applicable for elucidating the relationships between these features separately for different dimensions of QoL. The results contribute to a deeper understanding of the health-related situation of cancer patients and provide starting points for providing more fine-tuned psychosocial care. Future research should consider the distinction between met and unmet needs, and the relationship between the reported needs and the real utilization of psychosocial support services [50].

## Figures and Tables

**Table 1:** Sociodemographic and clinical characteristics of the sample (*n* = 1108).

	*n*	%
**Sex**		
Males	404	36.5
Females	704	63.5
**Age group**		
18–39 years	220	19.9
40–49 years	183	16.5
50–59 years	327	29.5
60–69 years	233	21.0
≥70 years	145	13.1
**Education ^a^**		
Elementary school (8–9 years)	248	22.4
Junior high school (10 years)	367	33.2
High school/university (≥11 years)	486	44.0
No formal qualification	4	0.4
**Employment status ^a^**		
Employed	703	63.7
Unemployed	43	3.9
Retired	282	25.5
Other	76	6.9
**Tumor localization** **(main diagnosis)**		
Breast	381	34.4
Gastrointestinal tract	171	15.4
Prostate	144	13.0
Hematological	131	11.8
Female genital organs	78	7.0
Thyroid/endocrine glands	36	3.2
Melanoma	26	2.3
Male genital organs	23	2.1
Other	118	10.6
**Secondary tumor**		
No	988	89.2
Yes	120	10.8
**Time since diagnosis ^a^**		
<6 months	334	30.2
6 months–<12 months	374	33.8
12 months–<24 months	251	22.7
≥24 months	148	13.4
**Treatment**		
**Surgery ^a^**		
No	121	10.9
Yes	986	89.1
**Chemotherapy ^a^**		
No	508	46.1
Yes	595	53.9
**Radiotherapy ^a^**		
No	538	50.2
Yes	534	49.8
**Hormone therapy ^a^**		
No	780	74.0
Yes	274	26.0
**Antibody therapy ^a^**		
No	864	82.8
Yes	179	17.2

^a^ Missing data not reported.

**Table 2:** SCNs, satisfaction, and importance ratings (range: 1–5).

		Phys. Funct.	Autonomy	Emot. Stability	Cogn. Funct.	Social Relat.	Vitality	Absence of Pain	Sleep Quality	Global Health
SCNs	M	3.41	2.07	3.11	2.84	1.88	3.10	2.93	2.93	3.45
	(SD)	(1.06)	(1.21)	(1.28)	(1.28)	(1.15)	(1.15)	(1.37)	(1.39)	(1.09)
Satisfaction	M	2.84	4.06	3.29	3.10	4.14	3.06	3.61	2.95	3.25
	(SD)	(1.03)	(0.95)	(1.02)	(1.07)	(0.88)	(0.96)	(1.12)	(1.18)	(0.92)
Importance	M	4.03	4.51	4.30	4.19	4.43	4.12	4.35	4.25	4.43
	(SD)	(0.65)	(0.63)	(0.58)	(0.62)	(0.63)	(0.62)	(0.67)	(0.62)	(0.57)
Satisfaction change	M	2.02	2.81	2.40	2.25	3.20	2.27	2.64	2.31	2.30
	(SD)	(1.06)	(0.87)	(0.99)	(0.92)	(0.92)	(0.96)	(1.01)	(1.00)	(1.06)
Importance change	M	3.88	3.68	4.04	3.79	3.92	3.96	3.90	3.97	4.39
	(SD)	(0.96)	(0.89)	(0.81)	(0.85)	(0.90)	(0.79)	(0.89)	(0.84)	(0.73)

**Table 3:** Pearson correlations between SCNs and other variables.

	Phys. Funct.	Auto-Nomy	Emot. Stability	Cogn. Funct.	Social Relat.	Vitality	Absence of Pain	Sleep Quality	Global Health
SCNs—Satisfaction	−0.32 ***	−0.48 ***	−0.53 ***	−0.58 ***	−0.34 ***	−0.46 ***	−0.53 ***	−0.66 ***	−0.40 ***
SCNs—Importance	0.11 ***	−0.19 ***	0.12 ***	0.10 ***	−0.08 ***	0.12 ***	0.08 ***	0.20 ***	0.19 ***
SCNs—Satisfaction change	−0.22 ***	−0.28 ***	−0.37 ***	−0.47 ***	−0.15 ***	−0.35 ***	−0.38 ***	−0.48 ***	−0.26 ***
SCNs—Importance change	0.21 ***	0.24 ***	0.39 ***	0.42 ***	0.21 ***	0.32 ***	0.37 ***	0.48 ***	0.33 ***

***: *p* < 0.001.

**Table 4:** SCNs broken down by sex, age group, and tumor type.

		Phys. Funct.	Auto-Nomy	Emot. Stability	Cogn. Funct.	Social Relat.	Vitality	Absence of Pain	Sleep Quality	Global Health
**Sex**										
Males	M	3.37	2.21	2.83	2.62	1.98	2.96	2.80	2.77	3.32
	(SD)	(1.05)	(1.26)	(1.28)	(1.28)	(1.18)	(1.16)	(1.35)	(1.40)	(1.12)
Females	M	3.44	1.99	3.27	2.97	1.82	3.18	3.01	3.02	3.53
	(SD)	(1.06)	(1.17)	(1.26)	(1.26)	(1.13)	(1.14)	(1.38)	(1.38)	(1.06)
Effect size		0.07	−0.18	0.35	0.28	−0.14	0.19	0.15	0.18	0.19
**Age group**										
≤59 years	M	3.48	2.00	3.31	2.99	1.85	3.19	3.06	3.07	3.59
	(SD)	(1.04)	(1.18)	(1.26)	(1.25)	(1.10)	(1.14)	(1.36)	(1.36)	(1.06)
≥60 years	M	3.29	2.20	2.71	2.56	1.93	2.92	2.68	2.65	3.18
	(SD)	(1.09)	(1.25)	(1.24)	(1.28)	(1.25)	(1.16)	(1.38)	(1.41)	(1.10)
Effect size		−0.18	0.16	−0.48	−0.34	0.07	−0.23	−0.28	−0.30	−0.38
**ANOVA Significance *p***										
Sex		0.401	0.058	0.001	0.001	0.045	0.022	0.071	0.100	0.085
Age		0.006	0.096	0.001	0.001	0.468	0.002	0.001	0.001	0.001
Interaction Sex * Age		0.157	0.105	0.978	0.492	0.852	0.684	0.157	0.753	0.932
**Tumor type**										
Breast	M	3.42	1.86	3.26	3.05	1.79	3.14	2.92	3.02	3.50
(*n* = 381)	(SD)	(1.09)	(1.09)	(1.29)	(1.27)	(1.09)	(1.19)	(1.36)	(1.37)	(1.08)
Gastrointestinal tract	M	3.50	2.31	2.91	2.69	1.86	3.18	3.05	2.94	3.53
(*n* = 171)	(SD)	(1.08)	(1.32)	(1.26)	(1.21)	(1.11)	(1.08)	(1.36)	(1.39)	(1.06)
Prostate	M	3.12	2.08	2.63	2.44	1.90	2.71	2.62	2.57	3.11
(*n* = 144)	(SD)	(1.09)	(1.26)	(1.24)	(1.32)	(1.19)	(1.18)	(1.38)	(1.41)	(1.15)
Hematological	M	3.56	2.03	3.09	2.86	2.01	3.30	2.91	2.81	3.51
(*n* = 131)	(SD)	(0.97)	(1.24)	(1.35)	(1.33)	(1.26)	(1.15)	(1.33)	(1.47)	(1.11)
Female genital organs	M	3.56	2.29	3.42	2.94	1.77	3.15	3.12	3.01	3.53
(*n* = 78)	(SD)	(0.96)	(1.26)	(1.23)	(1.26)	(1.09)	(1.09)	(1.50)	(1.40)	(1.04)
Thyroid/endocrine glands	M	3.39	1.86	3.28	3.22	1.80	3.39	2.78	3.28	3.47
(*n* = 36)	(SD)	(0.90)	(1.05)	(1.28)	(1.22)	(1.08)	(0.96)	(1.46)	(1.26)	(1.03)
Melanoma	M	3.44	2.19	3.56	2.88	2.19	3.28	3.04	3.35	3.58
(*n* = 26)	(SD)	(0.82)	(1.13)	(1.16)	(1.24)	(1.30)	(1.02)	(1.31)	(1.26)	(0.95)
Male genital organs	M	3.59	1.95	2.77	2.64	1.86	2.50	2.55	2.82	3.14
(*n* = 23)	(SD)	(1.05)	(1.05)	(1.48)	(1.40)	(1.08)	(1.30)	(1.40)	(1.33)	(1.32)
Others	M	3.35	2.33	3.20	2.70	2.01	3.08	3.19	2.98	3.50
(*n* = 118)	(SD)	(1.07)	(1.23)	(1.23)	(1.17)	(1.26)	(1.08)	(1.30)	(1.35)	(1.04)
ANOVA significance *p*		0.028	0.001	0.001	0.001	0.423	0.001	0.031	0.026	0.015

## Data Availability

Raw data supporting the conclusions of this article will be made available by the corresponding author on reasonable request.
